# Enabling Reuse in Extended Producer Responsibility Schemes for White Goods: Legal and Organisational Conditions for Connecting Resource Flows and Actors

**DOI:** 10.1007/s43615-021-00053-w

**Published:** 2021-05-12

**Authors:** Carl Dalhammar, Emelie Wihlborg, Leonidas Milios, Jessika Luth Richter, Sahra Svensson-Höglund, Jennifer Russell, Åke Thidell

**Affiliations:** 1 IIIEE, Lund University, PO Box 196, 221 00 Lund, Sweden; 2 Department of Sustainable Biomaterials, Virginia Polytechnic Institute & State University, 1650 Research Center Drive, Blacksburg, VA 2406 USA

**Keywords:** Circular economy, Extended producer responsibility, Product policy, Reuse, Repair, Refurbishment

## Abstract

Extended producer responsibility (EPR) schemes have proliferated across Europe and other parts of the world in recent years and have contributed to increasing material and energy recovery from waste streams. Currently, EPR schemes do not provide sufficient incentives for moving towards the higher levels of the waste hierarchy, e.g. by reducing the amounts of waste through incentivising the design of products with longer lifespans and by enhancing reuse activities through easier collection and repair of end-of-life products. Nevertheless, several municipalities and regional actors around Europe are increasingly promoting reuse activities through a variety of initiatives. Furthermore, even in the absence of legal drivers, many producer responsibility organisations (PROs), who execute their members’ responsibilities in EPR schemes, are considering promoting reuse and have initiated a number of pilot projects. A product group that has been identified as having high commercial potential for reuse is white goods, but the development of large-scale reuse of white goods seems unlikely unless a series of legal and organisational barriers are effectively addressed. Through an empirical investigation with relevant stakeholders, based on interviews, and the analysis of two case studies of PROs that developed criteria for allowing reusers to access their end-of-life white goods, this contribution presents insights on drivers and barriers for the repair and reuse of white goods in EPR schemes and discusses potential interventions that could facilitate the upscale of reuse activities. Concluding, although the reuse potential for white goods is high, the analysis highlights the currently insufficient policy landscape for incentivising reuse and the need for additional interventions to make reuse feasible as a mainstream enterprise.

## Introduction

A key strategy for reaching a circular economy (CE) is to keep the value of materials, products and components at their highest utility level [1]. In other words, it is important to avoid the wasteful practice of producing poor-quality, short-lived products and materials. Therefore, in a CE, actors of key importance include all the stakeholders that engage with activities that prolong the lifespans of products, such as manufacturers of high-quality products and product-service offerings, companies providing maintenance and service activities, repairers and remanufacturers and firms selling reused products, components and materials. Some business actors, for instance those engaged in remanufacturing operations, have been an important contributor in extending product life in high-value industrial sectors (e.g. aerospace, locomotive, heavy-equipment), while similar business engagement is growing fast in consumer-facing sectors including vehicle parts, ICT and office furniture [2, 3]. Several municipalities and regional actors around Europe are increasingly promoting reuse activities through a variety of initiatives, e.g. by introducing reuse infrastructure and second-hand commercial outlets in conjunction with municipal recycling centres (i.e. reuse parks) [4, 5].

### Overview of the Policy Landscape for Repair, Refurbishing and Reuse

There is a growing interest among policymakers in promoting both higher-quality products and various reuse and repair activities that can prolong the lifetime of products. Policymakers at various levels of government have adopted or proposed a variety of policy measures relevant to product life extension (cf. Table 1).

**Table 1:** Examples of adopted and proposed policies to increase product lifetimes (amended version of table in [6]. *A* adopted measures, *P* proposed measures)

	European Union	EU Member States	Other (local/regional)
Adopted	*Ecodesign Directive*: new mandatory requirements on products put on the EU market; related to durability, reparability, provision spare parts etc. *Standardisation* activities to develop new product standards on concepts such as ‘durability’, ‘reuse’, ‘reparability’ and ‘recyclability’; will make it easier to regulate these issues in future laws (ongoing process)	*Criminalising planned obsolescence* (France) *Fines for planned obsolescence* (competition authority of Italy) Strengthening *legal* (mandatory) *product guarantees* in consumer law (several EU countries) *Tax reliefs for repair* (e.g. Sweden) *National accreditation* of reuse organisations (e.g. Belgium) *Repairability index* (France)	*Public procurement* of remanufactured ICT and furniture (e.g. Sweden) *Reuse parks* and similar infrastructure; diverting EOL products towards reuse *Networks for reuse*, including infrastructure, quality controls and marketing (e.g. the Flemish reuse network) and repair networks (e.g. Vienna) *Encouraged use of remanufactured spare parts* for federal government vehicle fleet maintenance (e.g. USA) *Government support for private reuse firms* (e.g. Sweden) *Quality labels for reused goods* to instil consumer confidence (several EU countries)
Proposed	*Consumer law* changes to ensure that consumers receive trustworthy information on product lifespan, the availability of repair services, spare parts and repair manuals *Measures to promote right-to-repair (R2R)* *Public procurement criteria* for remanufactured goods	*National public procurement criteria* for remanufactured goods like furniture and ICT products (under development) *Standards and quality labelling* schemes for reused products (under development)	*Right-to-repair (R2R) laws* proposed in several US states; including several provisions to enable consumers to repair their products and allow independent repairers to access the after-sales market

Table 1 shows an emerging ‘new generation’ of policies that make use of quite different incentives—aimed at different actors—to prolong product lifetimes and service lives. The manifold policy interventions are essential due to the gravity of the existing ‘linear’ paradigm in economic activities, making it hard for companies with a circular business model to compete on the market [7, 8]. Current tax schemes, regulations, supply chains and consumer habits are all ‘geared’ into linearity, and thus new types of solutions face a multitude of institutional barriers [9]. A number of existing policies and laws can act as barriers to the establishment and scale-up of circular businesses [7, 8]. Within the EU, rules related to chemicals and waste constitute impediments for reuse and repair in various ways [6, 10–12]. Recent efforts by the European Commission are aiming to address these issues, to the extent possible, in order to accelerate the transition to a CE [13, 14].

### Objective and Structure of This Paper

Although a general understanding of the barriers, drivers and policy tools to enable repair, refurbishment and reuse of products is beginning to emerge [1, 15, 16], there is a need to apply these lessons to more specific contexts and identify the contextual challenges of promoting the preparation for reuse in different product groups and various organisational settings. This paper focuses specifically on the case of preparation for reuse of white goods from producer responsibility organisation (PRO) waste streams, including repair, refurbishment and reuse operations of end-of-life (EOL) products in the wider setting of extended producer responsibility (EPR) schemes. White goods are among the consumer waste streams with the highest potential for reuse [4, 17, 18], and therefore it serves as an illustrative case to identify the organisational challenges and the required policy interventions. The case study of white goods in EPR schemes will facilitate a comprehensive understanding of the specific conditions for preparation of reuse of discarded appliances in light of research on more general context of repair, refurbishments and reuse.

Therefore, the objective of this paper is to determine strategic actions for upscaling the preparation for reuse of discarded—PRO-controlled—appliances, by identifying the barriers and drivers and actor interactions, in order to suggest the type of policy measures that can enable such an upscale. The chosen product group of study is white goods. To this effect, we seek to create a comprehensive understanding of the legal and organisational barriers and drivers, particularly from the perspective of the various actors and their interests in the preparation for reuse.

The next section presents a brief overview of the literature on the potential for reuse of white goods, including a background section on extended producer responsibility and reuse and preparation for reuse activities. It then analyses the potential for reuse of white goods and barriers that hamper that potential, as well as drivers for increasing its implementation. The methodology of this study is presented next, followed by a section presenting the empirical findings from the interviews with relevant stakeholders in the reuse of white goods and the presentation of two case studies of PROs developing criteria for repairers wanting to access, repair and sell white goods from PRO-administered material streams. The article continues with the discussion of barriers and drivers identified from the analysis of the findings and case studies. Finally, the article ends with some concluding remarks.

## Literature Background

### Extended Producer Responsibility and Reuse

EPR policies allocate responsibilities to producers (and other actors) for the EOL management of their products while also intending to provide incentives for ecodesign (see [19]). Producers of electrical and electronic equipment (EEE) are required, according to the Waste Electrical and Electronic Equipment (WEEE) Directive 2012/19/EU [20], to ‘finance at least the collection from collection facilities, and the treatment, recovery and disposal of WEEE’ (Preamble (23) [20]). However, it is argued that EPR is not intended to simply allocate the financial responsibility for waste management but also to incentivise producers to implement waste reduction strategies upstream, such as reuse, repair and refurbishing [21]. Indeed, Article 4 of the WEEE Directive states that Member States shall ‘encourage cooperation between producers and recyclers and measures to promote the design and production of EEE, notably in view of facilitating reuse, dismantling and recovery of WEEE, its components and materials’.

Even though the WEEE Directive promotes reuse as well as recycling, it sets combined targets for preparation for reuse and recycling (e.g. Annex V, Part 2 and 3), leaving Member States and stakeholders (e.g. PROs) with the possibility of reaching these targets by recycling alone. Thus, although current legislation for EPR has contributed to increasing material and energy recovery of WEEE, it has generally failed to provide meaningful incentives for reducing the amount of waste and achieving higher levels of the waste hierarchy, e.g. product design to increase lifespan and/or the ease of repair [22, 23]. In essence, producers are merely supporting the financial burden of waste management and tend to disregard the potential of extending their business models to repair and reuse as waste reduction strategies [21].

Increasing the share of ‘preparation for reuse’ operations from the PRO-controlled waste stream would allow for WEEE to reach higher levels in the waste hierarchy. One issue is the fact that PROs are not getting access to all of their products at their EOL; instead, ‘undocumented WEEE’ are either disposed of in the mixed residual waste stream, held on to or passed on by consumers, scavenged for valuable materials and parts, or become exported [24]. This makes it all the more important that the WEEE that does enter the EPR streams goes through the most value retaining process.

### The Preparation for Reuse Process and Relevant Actors

**Fig. 1:**
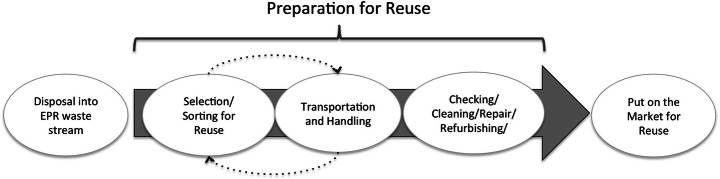
Preparation for reuse of white goods in EPR scheme

‘Reuse’ constitutes a waste prevention strategy in that, instead of becoming waste, the product is employed by a second user in the same manner as it was intended for. This is distinguished from ‘preparation for reuse’, which applies to reusable products that have entered the official waste stream. The process of preparation for reuse consists of several steps (Fig. 1). ‘Repair’ itself consists of the retaining or restoration of functionality, while ‘refurbishing’ involves the replacement of several major components of the device [1].

First, the waste must be disposed of by the consumer and collected in the EPR system. When the consumers dispose of an appliance at a designated collection facility, or handed it over directly to a PRO, the appliance is said to have entered into the EPR waste stream. It then becomes ‘waste’ according to the WEEE Directive and, depending on the setup of the EPR system, can then be seen as the property of the producer or PRO obligated to take care of this waste.

The reusable waste may need to be separated from other waste if collected together and may need to be transported for the next step. Preparation for reuse of this waste entails ‘checking, cleaning or repairing recovery operations, by which products or components of products that have become waste are prepared so that they can be reused without any other pre-processing’ (Directive 2008/98/EC). Preparation for reuse implies that after quality check, including repair if necessary, a useable good does not belong in the waste regime anymore and can be sold or donated to consumers [25] or sold to professional remanufacturers or similar.

According to the WEEE Directive, ‘reuse organisations’ have the right to access the EPR waste streams to prepare waste for reuse, but there is little guidance on how this should be decided, and there is still ambiguity and disputes about who is entitled to access waste [26]. For instance, in Sweden, municipalities have the responsibility to collect, transport and recycle household waste (15 Chapter 20 § MB (Miljöbalk SFS 1998:808)). However, the waste at the collection site that belongs to the PRO is managed by them. If the PRO chooses to do so, the appliances with reuse potential move toward such processing, which is handled either by a specific branch of the PRO or the batch is handed over to a third party.

Regardless of who is in charge of the processing, the waste products are essentially put through a similar process; they are first further assessed and then prepared for reuse (i.e. cleaning, or sometimes also minor repairs), while some appliances need more thorough refurbishing in order to be brought back into working order. There is a European standard for ‘Requirements for the preparation for reuse of waste electrical and electronic equipment’ (EN 50614). Once the device is in working order, it is put on the market for reuse.

The PROs gain possession of the appliance at the moment of disposal. The PROs fulfil the WEEE directive on behalf of the producers. This means, in theory, that they have an obligation to work with Member States to achieve the reuse and waste prevention objectives in the WEEE Directive. In practice, PROs often contract third party actors for collection (e.g. with municipal waste management companies), transportation and recycling of the discarded products.

There are also third parties with potential interests to access the PRO-controlled waste streams, such as ‘gap-exploiters’ (i.e. reuse and refurbishing companies—see [12]), municipalities, social enterprises and non-profits. Further, actors, such as repairers and reuse organisations, are interested in accessing the PRO-controlled waste streams to either recuperate entire appliances or strip them of reusable spares.

With regard to the sellers of reused products, many of the economic actors engaged in remanufacturing and refurbishment utilise global networks to distribute their finished products into markets around the world and manage reverse-logistics systems to recover valuable product modules and components for use in remanufacturing activities [1]. The consumer demand for refurbished equipment—instead of new—dictates the profitability of the entire process of preparation for reuse [27]. Further, a critical aspect of a well-functioning reuse system is the willingness of consumers to engage in the return of old equipment in their possession [28, 29], although that stage is beyond the scope of this paper (see Fig. 1).

Several European PROs have started pilot projects in cooperation with other actors to test refurbishment business models for EOL products. In order to have any significant environmental impact, such pilot initiatives must be scaled up. However, the process of preparing appliances in EPR schemes for reuse is currently impeded by several legal, market and logistic-based barriers that need to be better understood, along with how they can be mitigated.

### Potential for Reuse of White Goods

White goods are among the consumer waste streams with the highest potential for reuse [4, 17, 18]. Often consumers are less interested in the aesthetics of such ‘workhorse’ products and more interested in value and price [30]. When reaching their EOL, a large share of discarded white goods can be found in relatively good condition, but in the case the product cannot be used in its entirety, then its components may still have a sufficient value for reuse [31]. Estimation of the reuse potential of different types of white goods varies, but a study on UK household WEEE concluded that ca. 26% of larger WEEE products can be re-sold directly, whereas another 23% could be reused after repairs and sold at a profit [32]. Other studies found this estimation to be a little optimistic and indicated smaller shares of reusable and repairable equipment but argued that this may depend upon the condition of the white goods as damages are sometimes inflicted during storage and transport [4, 17, 33].

### Barriers to Reuse of White Goods

#### Barriers in Collection, Transportation and Separation for Preparation for Reuse

The most common reason that a product is discarded is that it is broken, but this is not always the case, which explains why functioning products can be found at recycling stations [32, 34]. A Danish study found that relying on consumers to conduct the separation of reusable white goods at the waste collection site did not result in sufficient separation. Instead, experts physically present at these collection sites were needed in order to conduct proper assessment of the conditions of each item [26]. Most commonly, it is the municipalities that are in charge of the collection sites, but since they do not own the EPR-obligated appliances, there are poor incentives for municipal authorities to manage the site in a way that favours reuse. The PRO might not even examine the waste before transporting them from the collection site [35]. This is confirmed by a PRO report that states: ‘[f]inancially, there is no incentive to look for functioning products in the WEEE collection’ ([36] p. 20). Potential reuse organisations and actors who might have the incentive to separate and prepare products for reuse are often denied access in practice [37].

Several studies found that in the handling of discarded white goods, careless loading/unloading of appliances into collection containers took place regardless of the condition of the white goods or the damage that the rough treatment incurred onto these appliances [4, 17, 33, 38].

In a Danish study, Larsen et al. [39] investigated the roles and interests of a large number of players in the electronic equipment supply chain to, amongst other, identify challenges to prepare for reuse. A variety of challenges were identified, of which many were relevant to the collection, transportation and separation of discarded equipment. Specifically, it was reported that it is difficult to sort out and get access to high-quality products that are suited for repair and reuse. This challenge has several facets such as the logistics of bringing used products from consumers or businesses to the reuse facilities. The collected items are often damaged by handling at the collection points, and it takes professionalism and knowledge to repair products safety, as there is generally a lack of safety standards. Many of the above-mentioned challenges imply additional costs such as high labour costs, costs to build and assure knowledge and operations, initial costs for additional efforts in collection and building up the infrastructure, which ultimately reduce the profitability of operations from selling reused products.

Moreover, a number of other administrative issues are present, including the burden of data collection and reporting of handled products, assuring data security in memory units, and establishing and verifying compliance with operations and competence certification systems. It was also concluded that far from all used products were suitable for reuse due to high energy demand and content of hazardous or banned substances [39].

#### Barriers in Repair and Refurbishing as Part of Preparation for Reuse

Repair activities can be particularly challenging since there are many differences between various models and brands and a high turnover of new design solutions for white goods [38, 40]. Moreover, there may not be enough relevant repair information from producers [16]. Every product model has its own specifications and diagnostics, which means that repairers need to have access to information. If the components are specialised and not common, finding spare parts can be difficult, and often producers charge high prices for spare parts [38, 41]. In some cases, spare parts are only offered for a limited number of years [40]. Producers may also discourage independent repairers by offering tools and diagnostic software only to certain, authorised certified repairers [16]. Therefore, the cost of repair, access to spare parts and knowledge about repair options can be a barrier to repair [31, 32, 42].

Product design also plays a critical role in the repairability of a specific product. If the product’s design hinders the access and replacement of integral components, then it reduces the potential for reuse and repairs [16, 38, 43].

Finding skilled and experienced repairers of appliances is another challenge for repair actors [38]. The low profitability in the industry makes it difficult to attract and retain highly skilled repairers [27]. Producers are concerned about the quality of repairs and generally prefer that their products are repaired by their authorised or recommended repairers [16, 44]. Having skilled repairers constitutes a must, as unskilled repairers have been found to not be able to conduct satisfactory repairs even with access to manuals [37].

However, some of the challenges to repair are related to the organisation of the EOL product management system and the interactions between the actors. Larsen et al. [39] refer to low trust and weak collaboration between actors and other conflicting interests between the actors as critical barriers. The conflicts could be related to distortion of recycling targets, distribution of reuse related costs and revenues between actors but also brands considering risks of bad reputation from uncontrolled reuse and reselling of their products. The latter is related to a fear of poor consumer perceptions and acceptance of buying used and repaired products, which also limits their market.

#### Barriers to Returning Reused Products to the Market

Further, there is a wide variety of barriers that affect directly and indirectly repairs, including laws related to intellectual property rights, as well as contract law and consumer law that can be used by manufacturers to impede repair activities by consumers and non-affiliated repairers; these are in addition to market barriers, such as the low price in replacement products and conflicting consumer preferences [16].

Consumers have hard-embedded attitudes and habits towards existing products and market offerings, which often constrain their willingness to buy reused products [29]. Some consumer groups are also sensitive to issues such as fashion trends and product appearance and prefer products that have a new look, which makes them averse to reused products. Moreover, the consumers’ willingness to pay for reused products differentiates with regard to the perceived risk that the products are of lesser quality [44]. Hence, measures such as quality labels for reused products can be used to instil consumer confidence [45]. One study found that 37% of Europeans are interested in purchasing second-hand EEE [42].

Risk of lower-quality reused products can also pose a risk to producers in terms of brand risk [46]. The potential of reused products to cannibalise on the sales of new products has also been viewed as a possible barrier to motivating producers towards reuse [46].

For exporters of refurbished goods, trade laws and other regulatory mechanisms that govern the movement of remanufactured and refurbished goods across borders significantly affect the extent to which these can be exported [1]. Many countries are concerned about the ‘dumping’ of waste disguised as refurbished or remanufactured export goods and impose restrictions or prohibitive requirements (e.g. fees, paperwork, additional inspections) on these imported goods. These measures can effectively reduce the competitiveness of ‘circular goods’ in the marketplace, relative to new ones [47].

Further, there are legal challenges grounded in terminology and definitions. This can be attributed either to the presence of explicit language that imposes onerous operational friction for companies trying to engage in circular economy or the absence of clearly differentiated definitions which results in confusion, lack of clarity and incompatibility with circular economy activities. For instance, in Brazil, the government does not distinguish between ‘used’ goods and multiple service life goods (e.g. finished remanufactured products) in its import laws [2]. Thus, refurbished goods are effectively prohibited because they are not meaningfully differentiated from ‘used’ goods and are governed by the same import laws that were instituted to prevent the dumping of outdated technology [47, 48].

### Drivers for Reuse

#### Measures to Improve Preparation for Reuse from Waste Streams

By optimising the collection system, damages to products due to handling and transportation can be avoided or significantly reduced. This could be done by having a clear separation between collection for reuse vs. waste for other treatments [38]. Moreover, routines for handling, storage and transport must be adapted to protect goods with reuse potential. This could be accomplished by providing reuse actors access to the products as soon as possible, before the appliances enter the WEEE stream [38].

Regarding economic potential for reuse, access to large volumes is probably necessary for profitable business [17]. This also means that preparation for reuse seldom has high economic potential for OEMs with small volumes. Collaborations with actors with existing structures could be a way to reduce costs [17]. The social sustainability aspect and job creation in preparation for reuse and repair is an important factor [49] that could drive producer engagement. Further, especially for producers, engaging in preparation for reuse efforts can reduce the overall environmental impacts of the producer [17].

Drivers such as an open dialogue between manufactures and the reuse organisations involved in preparation for reuse, policies favouring social activities, municipal support for reuse, funding and education have also been identified in an EU Commission study examining the possibility of introducing reuse targets in the WEEE Directive [50]. Some Member States are already experimenting with policies for reuse. Spain, for example, has introduced targets for reuse of WEEE. For white goods, the target started at 2% and rose to 3% in 2018 [43]. Such quotas have been suggested as important drivers for increased reuse of WEEE [17]. Inclusion of reuse in reporting of recycling rates is also deemed crucial for upscaling preparation for reuse [26].

#### Measures to Improve the Conditions for Repair and Refurbishing

Several proposals for improving the conditions for repair are present in literature include mandating that producers provide spare parts for a number of years after a product is launched on the market [40, 51], improving the harvesting of functioning parts for use as spare parts [51], changing the design of products so as to become more modular and easier to disassemble [31] and the standardisation of components [31]. In the EU, such measures are taken through the new Ecodesign Directive Regulations which were recently adopted, whereas in the US, new proposed R2R laws address some of the issues [16]. While lack of information for repair is a barrier to repair generally, according to Article 15 of the WEEE Directive, producers are obligated to provide information on the product to the waste management actors for preparation for reuse (i.e. repair). Enforcement of this requirement could address a general repair barrier for at least the segment of repairable products that are discarded and are part of the EPR system.

To address the consumers’ willingness to purchase reused products, several policies have been promoted in literature, including the provision of information about the ecological potential of repair and reuse [34] and quality labelling for reused goods to instil consumer confidence [45].

## Methodology

The article departs from a preliminary literature review on the subject of reuse and repair for white goods (in ‘Literature Background’), particularly related to the WEEE stream and the EPR operations around it, and focusing on the identified barriers and drivers for reuse and repair operations in literature.

The literature findings constituted the subject background for an interview study with actors immediately involved in reuse and repair operations of white goods in the Nordic countries. The literature informed the direction of the interview study and facilitated the development of the interview protocol which formed the basis of the semi-structured interviews.

Moreover, draft guidelines issued by a Nordic PRO, which are currently under development, were also analysed. These guidelines are quite unique as they constitute—to the best of our knowledge—the first time a PRO has set up written criteria for reuse. The guidelines describe in detail specific criteria that the PRO is planning to introduce to facilitate higher reuse in EPR WEEE streams; it aims to support reusers who want to get access to the PRO’s EOL streams while also safeguarding the interests of the OEMs that are members of the PRO. In order to allow reuse of their products, OEMs want certain safeguards regarding, e.g. product recalls and product liability issues. There are currently no adopted guidelines in place in the Nordic countries, but in interviews with other PROs, we have learned that they are considering developing guidelines with a similar content.

**Fig. 2:**
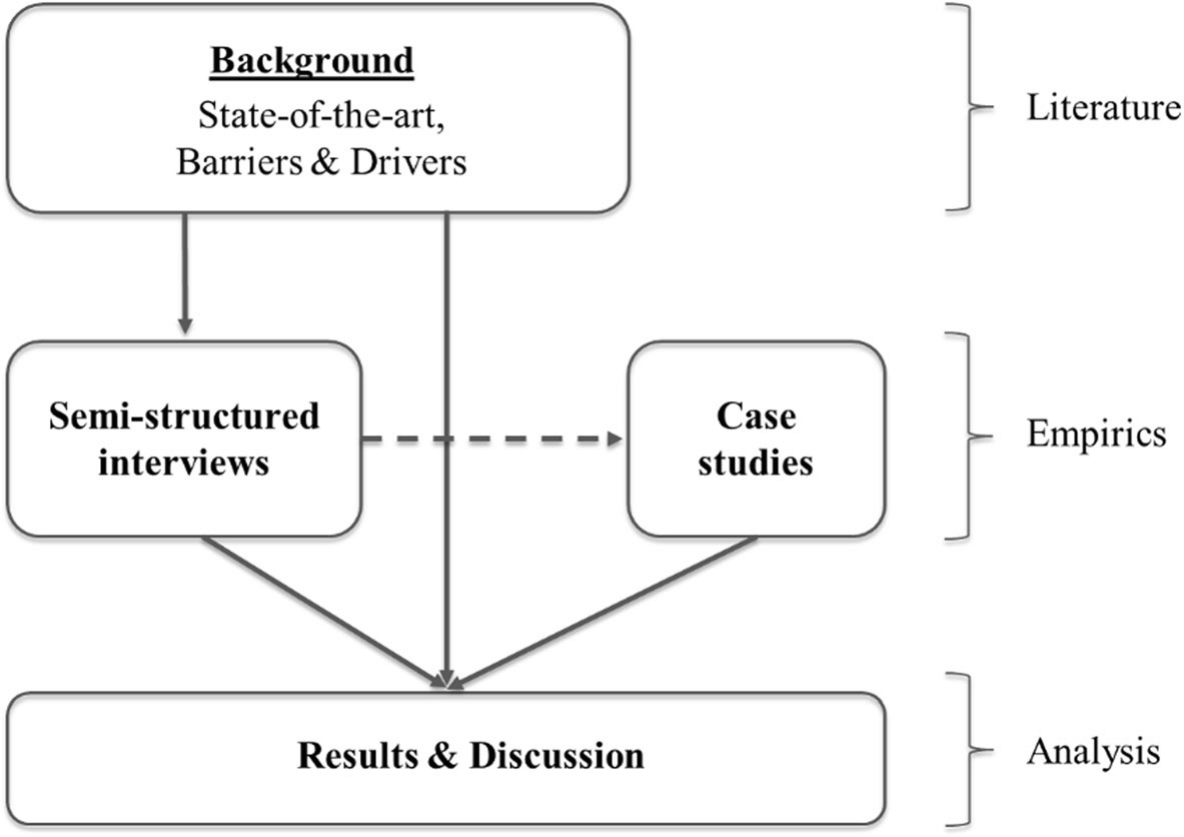
Methodology flowchart showing the main steps of the research process

The flow of the research process is depicted in Fig. 2.

To address the objectives of this study, a qualitative analytical approach was considered most appropriate, since it places greater emphasis on how individuals interpret and perceive reality [52], which provides more nuanced and detailed information [53] about the subject of the study. Semi-structured interviews were suitable to provide an in-depth insight into barriers and drivers of reuse of white goods for various players in the industry, and the preliminary literature review was appropriate to draw a background of how the different parts of the subject are reflected in existing research so far.

### Background Literature Review

The purpose of the background literature review was to anchor the present contribution into what is known on the barriers and drivers of repair, refurbishment and reuse and to direct the interview study, as well as enhancing the analysis of the interviews. Both academic literature and documents from authorities and relevant actors were sought for, in order to combine knowledge on the state of the art on WEEE reuse and which laws and regulations apply. To retrieve relevant literature, the search commenced on Web of Science, Google Scholar and LubSearch (Lund University library search engine). Keywords used include ‘circular economy AND extended producer responsibility’, ‘eco-design AND policy’, ‘household appliances AND repair/reuse’, ‘right to repair’, ‘WEEE AND repair/reuse’, ‘preparation for reuse AND WEEE’. Searches for documents from authorities and other relevant actors have been done via Google’s search engine. Additionally, the snowball method was used to identify relevant references, after the review of the first batch of search results. The snowball method is an inductive method and is useful for finding relevant sources within the same context [52]. The geographical scope was limited to the Nordic countries and Europe.

### Interview Study

To obtain empirical data from relevant stakeholders, semi-structured interviews were designed, based on the information gathered by the literature review, as a way to get nuanced insights into how the stakeholders view the topic, both in terms of opportunities, obstacles and future prospects. This means that the interview protocol followed relevant themes from the literature, including the identified barriers and opportunities, and expanded with open-ended questions to allow the interviewees to bring up additional information in the related topics and to propose potential solutions according to their experiences [52]. The main themes in the interview protocol were current white goods repair and reuse practices, expectations on market evolution in the future and various barriers and drivers. The questions about barriers and drivers related to various themes include current and future regulation and its implications and market-related issues (e.g. consumers and consumer attitudes, requirements for consumer warranties and current competition in the market). The interviews were conducted face to face or by telephone/video call. The interviews followed respective ‘consent and confidentiality’ requirements according to the interviewees preferences.

For the selection of respondents, the ‘snowball method’ and ‘appropriate selection’ were used. The term appropriate selection [52] means that the investigation of relevant actors to interview starts with contacting those that already have documented knowledge in the area of the prospective study. After the first round of contacts with relevant stakeholders who are knowledgeable in the field, the snowball method is applied, in which the contacted individuals indicate the most relevant actors that are actively engaged with activities in the area of the study and have sufficient knowledge to share with the researchers. In the present study, 14 relevant actors were identified and contacted for an interview, and only eight did return the interview request. Valid constraints for materialising all planned interviews included issues related to trade secrets and confidentiality, as well as unavailability due to the ongoing pandemic (COVID-19 virus) as of 2020. The interviews conducted included PRO organisations (3 interviewees), repair and reuse enterprises (2 interviewees), academia (2 interviewees) and finally one interviewee from a white goods producer (OEM). The geographical location of the interviewed actors is Scandinavia, where the relevant actors have shown keen interest in incentivising the repair/reuse of WEEE from EPR systems, strongly motivated by the increasing CE interest in the business and policy domains in the region.

The analysis of the interviews included the transcription and systematisation of the material so that it became possible to make interpretations from the information and discern the various perspectives that have emerged [53]. The material was coded according to emerging topical categories, and then each sub-category was assigned to either a barrier or driver of reuse operations as identified in the background literature. The full array of data categorisation is presented in the results section.

### Case Studies

During the interview with a PRO representative, the research team was provided with background information about documents being developed by the PRO, which examine criteria for reuse companies who want to access EOL products from the EPR waste stream. They were also provided access to the documents (elaborate draft guidelines). Final adoption of the guidelines is expected in the near future. Compliance with the guidelines will be necessary for any reuse organisation who wants to gain access to EOL streams handled by the PRO, and the PRO will monitor compliance and make audits. Therefore, a separate section of the results is dedicated to present and analyse the requirements brought forth by the PRO for how they affect third party access to the PRO waste stream. It is our understanding that PROs in other Nordic countries are considering similar guidelines, and the draft guidelines can exemplify the issues that PROs need to deal with if they provide third party reusers access to their EOL streams while also safeguarding the interests of the producers (OEMs). Further, based on interviews and documents with the Norwegian PRO Norsirk, we also account for their efforts to promote reuse, including cooperation with reuse organisations.

The main reason for including these cases are that the document obtained in the first case is quite unique and provides insight into key issues related to the ‘balancing of interests’ when allowing reusers access to EPR flows. The Norsirk case is included because it provides some insight into how PROs look upon future drivers for reuse. The cases constitute actual examples of practical applications of the issues elaborated in literature and provide the necessary background for developing further operational standards for reuse in PRO settings for the future. Therefore, the case studies provided invaluable context to the analysis and complemented the background literature and interview findings.

## Results

The results of the interview study (section 4.1) are divided into two sub-sections: barriers and drivers. Then, we present in detail the case studies (section 4.2), outlining the proposed criteria that PROs are planning to implement in relation to WEEE streams for enabling further reuse activities.

### Interview Study

Following the objectives of the study, the main elements of interest were the identification of barriers and drivers for actors involved in reuse operations and bringing forward the perspectives of actors concerning the potential future development of reuse, especially related to WEEE in the possession of PROs. Also, issues related to existing rules and regulations affecting the potential of reuse were highlighted. The results from the eight interviews are presented in detail in the following sub-sections, divided into barriers and drivers for collection, repair, refurbishment and reuse of EOL products.

#### Barriers

The interviewees identified an array of existing barriers spanning issues related to the design of products, consumer behaviour, the economics of repair, the unfit collection and handling operations in current EPR schemes and generally a suboptimal design of the framework of EPR which does not incentivise the repair and reuse of EOL products.

##### Barriers in Collection, Transportation and Separation for Preparation for Reuse

The respondents identified a general difficulty for OEMs to become interested and start working with the reuse of their products. The streamlined business plans of OEMs are optimised for a linear economy, and thus they lack the organisational capacity to develop reuse capabilities. Moreover, OEMs are concerned about cannibalisation in the sales of new products, as reuse would be taking up an increasingly larger share of their market.

There is no willingness among PROs to make funds available, or contribute, to reuse operations of their EOL products; hence, these operations must be profitable without such support. A major parameter that contributes to profitable reuse operations is the access to large volumes of EOL products without significant costs, or as one actor mentioned: ‘we need to get the white goods for free’. Reuse actors have unavoidable incurred costs related to labour hours (i.e. repair is a labour intensive activity), transport of large volumes and weight of EOL white goods (e.g. fridges, kitchen ovens etc.) and high warehousing requirements (spatially and temporally). In addition, there is a need for knowhow on what products are worth repairing and how to repair and test the repaired products. On the other hand, the sale prices are pushed down due to market competition and consumer preferences; thus, the profit margin is low. And this is why it is important to have access to the highest possible amount of EOL products at the lowest possible cost. One actor explicitly mentioned keeping costs low as a main barrier. So, it may be hard in reality for reuse actors to reach a sustainable profit margin, and one respondent claimed that reuse activities must therefore be combined with other business activities or partly paid through job training for socially vulnerable persons.

The majority of interviewees identified that a key barrier to reuse of white goods is stemming from the fact that current EPR systems tend to focus on recycling and thus fail to explicitly state targets for reuse, which results in infrastructure and incentives primarily organised for achieving higher material recycling. The fact that there is no reuse target in legislation, or at least a minimum reuse rate in the requirements of EPR schemes, makes it easier (and cost efficient) to focus exclusively on recycling. There is no part of the collection system that has a responsibility to ensure that reuse takes place, and this often means that fully functioning white goods end up into the WEEE stream for recycling.

Some respondents claimed that to safeguard the reuse potential of products, there might be two collection and logistics channels to improve handling and make it easier to distinguish between what is to be reused and recycled. Appliances that have potential for reuse must be protected during transport and during storage. However, there were counter arguments that it would be quite expensive to have two logistics lines. Alternatively, reuse actors could get access to the EOL equipment early in the collection system, before products are bundled for recycling, thus treating this EOL stream in a cascading manner prioritising reuse and then recycling and other recovery operations. However, the implementation of this dual system might be less likely due to the relatively higher costs of maintaining two separate EOL streams. Moreover, one respondent also mentioned the improvement of recycling technology that could make recycling more attractive in the future.

##### Barriers in Repair and Refurbishing During Preparation for Reuse

An important parameter which significantly hinders repair is the general lack of repair information and the limited availability of spare parts. A few respondents also mentioned that certain products require non-standardised repair tools and specific components, although the logic behind these repair-impeding product features are not obvious. Therefore, greater standardisation of components and fixtures would enable a universal application of repair services, with non-specific tools and without specific knowhow of each individual product.

##### Barriers to Returning Prepared for Reuse Products to the Market

According to the respondents, many producers are worried about the responsibility they may retain for the reused products when they enter a second (or third, etc.) time on the market. The fact that reused products are resold by other actors not controlled by the OEMs has led to concern about associated product liabilities. For instance, a reduced warranty period is typically provided for a reused product: a longer warranty is not justified due to the low price of the product. The consumer rights applied for new products could in some instances be difficult to apply for reused products. According to some interviewees, a reused product that carries the ‘CE mark’ (i.e. the mark signifying that the product meets all requirements and complies with EU product regulations, including safety standards) should be re-tested in order to show compliance in its second life; however this requires expensive tests.

According to the respondents, consumers are mainly interested in four aspects when considering purchasing a reused product: price, warranty, age and the brand. Consumers are not willing to pay a high price for reused products, equal or comparable to the minimum price of newly manufactured alternatives. Rather, the main reason for consumers to buy reused appliances, one interviewee said, is that it is much cheaper than the full price. At least 25% less cost is required compared to newly produced white goods. The segment of consumers buying reused products for environmental reasons is small. Moreover, the purchasing base of reused products consists often of consumers with limited income, and they would usually require a price discount for making the purchase (up to 50%, depending on circumstances, cf. to a new product).

In the experience of the respondents, consumers may perceive reused products as less trustworthy and/or having reduced quality due to wear and tear. The age and OEM brand of the reused product also makes a big difference to their willingness to purchase. The brand of the OEM that produced the white goods is therefore an important factor when consumers choose which product to buy. Certain OEMs have built a reputation of high-quality functioning products in contrast with cheaper alternatives of lower quality. This in turn means that the reusers who sell refurbished white goods of reputed brands can expect a higher turnover of these goods.

Further, the age of white goods is another factor that greatly influences the consumer’s willingness to buy. White goods that are only a few years old are more easily sold than those that are a few years older. Thus, to counterbalance consumer preferences, the price is respectively reduced for older products until a threshold of non-profitability (or cost) after which the reuser takes the decision to divert the product to recycling.

Most respondents mentioned that white goods are usually sold with a warranty of 6–9 months to be able to attract customers. There is a trade-off regarding the length of the warranty and the price of the product, as it is costly for the second-hand retailer or organisation to provide a warranty for the reused white goods. In order to offer longer warranties, reusers charge a higher price, making the reused white goods relatively less competitive.

#### Drivers

The interview study provided insights into what the driving forces are for engaging with reuse of white goods. The respondents highlighted that recently many companies have shown greater interest in promoting reuse, also OEMs.

##### Collection, Transportation and Separation for Preparation for Reuse

Several respondents suggested that reuse actually offers many benefits to the broader society (beyond the reuser and consumer), both environmentally and socially (e.g. job creation), and so it would be reasonable that the government supports it (e.g. with subsidies). Countries with high levels of reuse activities often have a strong social entrepreneurship agenda, and the social agenda associated with reuse was seen by the respondents as a way to increase OEM interest. Generally, the respondents also saw a large potential market for reuse, where there is yet limited competition, which means that it has the potential to attract more and more interest in the future. The EU Circular Economy Action Plan, and the proposed changes in policies and laws was seen as a driver for increasing the interest in ‘reuse business models’. Further, the respondents mentioned several policy interventions that could act as drivers for higher reuse, especially in the WEEE stream. For instance, the introduction of reuse quotas to complement existing recycling targets in the WEEE Directive. More stringent regulations are needed to discourage discarding and rough transport of EOL equipment that are in good condition. Another suggestion included the introduction of a deposit refund system for a selected group of products (e.g. white goods), in which a deposit is paid upon the purchase of the product from a retailer. At the EOL of the product, the consumer may redeem the deposit at the retailer and the appliance could be redirected for reuse or recycling upon assessment.

##### General Repair and Refurbishing as Part of Preparation for Reuse

The majority of respondents indicated that the new Ecodesign Directive requirements on design and spare parts may support future reuse but anticipated that it will take a long time before any significant effects become observable in the market. Moreover, the respondents identified the assignment of a clearer responsibility for the second life of products as a potential driver for reuse. For instance, there is uncertainty regarding if the preparation for reuse lies exclusively with the reuse commercial actors, or if OEMs could play a part as well. Clarifying this relationship would give a stronger mandate to the responsible actors to aim for higher reuse levels.

##### Returning Prepared for Reuse Products to the Market

A guarantee is important when selling reused products, as consumers want to feel secure in their purchase that a product will last a longer time. Therefore, the longer the warranty period offered, the higher the consumer interest. However, this can come with a trade-off of higher priced reused products, as noted in the ‘Barriers’ section above. Another way the producers can be incentivised to reuse their products could be through the offering of product-services instead of sales, which then makes it more feasible to reuse (also economically) and also allows them to maintain control over their products in subsequent life cycles. As they take back their products, it never becomes waste.

### Case Studies

#### PRO Pilot Project with Reuse Firm

A draft document currently being developed by a North European PRO examines criteria for reuse companies who want to access EOL products from an EPR waste stream. During a semi-structured interview in 2020, the PRO provided some background information about the document.

The PRO has, for the last couple of years, experienced that more and more actors have shown interest in accessing their EOL waste stream. This has included reuse/repair companies, as well as municipalities, who want to use them for repair workshops. The PRO and its member companies are positive towards making better use of the products and increasing reuse but have been concerned about several issues, such as the level of professionalism of the reusers, the way products are repaired and sold and the legal responsibility for repaired products. However, when representatives of the PRO visited one of the reuse firms, they were impressed by the level of professionalism of the company and started up a pilot project with them.

In this pilot project, the company can directly pick up white goods of their choice at a municipal recycling station; the recycling station is owned by a municipality, but the white goods that are handed in at the assigned area belong to the PRO. At the recycling station, citizens handing in white goods are met with a sign stating that their EOL product may be reused by the reuse company. This is to ensure that the EOL products are never considered to be ‘waste’ as soon as they are handed in. The reuse firm regularly visits the recycling station and picks up the white goods that contain adequate residual value according to their assessment. These white goods are repaired and refurbished (and/or harvested for spare parts) and are then exported to third countries (in the Balkan region of Europe) and sold there. The process of testing and refurbishing must however be performed at the country of origin of the EOL white goods, as export of electrical and electronic waste is usually not allowed according to EU law. The main reasons for selling them in a Balkan state is that it is much less costly to set up a retail outlet there; the rents and labour costs are significantly higher in Northern Europe, even if potential customers can be found there as well.

So far, the PRO is only working with one reuser in this pilot project, but they are getting an increasing number of requests from other reuse organisations. They are foreseeing a future development where they promote reuse by letting an increasing number of reusers obtain access to their EOL streams. This is something the PRO wants to do as they want to promote reuse, and their member companies (OEMs) are increasingly positive towards such developments. However, this process must be properly controlled. Therefore, the PRO is currently working on guidelines for all actors who want to have access to their EOL products. Compliance will be necessary for such access, and the PRO will monitor compliance and make audits. The main principle behind the guidelines is (1) to safeguard the interests of OEMs while (2) making it easier for reuse actors to engage with reuse, provided they follow proper procedure and with relevant documentation. Some of the key provisions in the guidelines, and an explanation for them, are provided in Table 2.

**Table 2:** Criteria for using EOL products from EPR streams

Criteria	Explanation
The product should not be more than 10 years old	Ensure there is no chemical content in the product banned under the RoHS Directive and other EU rules; and the product has been classified under the EU Energy label. Some exceptions possible for spare parts
Only products that have an established market and can be sold at a profit are accessed	The scheme is only open to professional reusers who operate commercially (not for, e.g. scrap recyclers and amateur repairers). The main requirement is that there should be a *market* for the reused products and that they should be sold at a *profit*
After reconditioning and repair, the products should be directly ready for reuse	The products must be prepared and tested properly regarding functionality, hygiene etc., so they can be directly sold and used. If the products fail certain specified tests, they must be handed over to recycling
Proper documentation and labelling	The products should contain information about the reconditioning organisation and information about testing and measures performed during reconditioning
Rules, export of reconditioned products	The products may only be exported to countries with proper recycling systems, and measures should be taken so the product is part of a PRO scheme
Passing of tests	Products that do not pass certain listed initial test procedures cannot be reused

The document particularly stresses that the reuse company must bear all the legal responsibilities for the product, e.g. according to all relevant EU product laws, when the reused product is sold to a consumer.

Further, the guidelines document lists certain indicators, based on the text of the Basel Convention [54], that provide an indication of whether a product in an EOL stream is appropriate to reuse or whether it should be ‘waste’ and go to recycling. Among the indicators listed, which would usually imply a product is ‘waste’ and not suitable for reuse, are missing vital parts, serious defects that hamper main functions and serious aesthetic defects that significantly impedes its commercial viability, and it contains dangerous substances that needs to be handled in a special way or is subject to export bans.

The guidelines have additional requirements and conditions. For instance, the PRO will set up a technical platform where OEMs may recall certain series of products that they deem not to be fit for reuse (e.g. due to inherent design issues). Further, the reusers must offer their customers a minimum warranty on the products. There are also requirements on transport of products for reuse, to ensure that the exported products are indeed not ‘waste’. This includes requirements on transport documentation, visual inspections of products and proper labelling and packaging.

The PRO wants to have guidelines that are clear and do not lead to unnecessary costs for reusers while at the same time securing the interests of its member companies and ensuring the PRO is in control of the flows. While the PRO’s member companies may be concerned about how products are treated, and the brand reputation risks involved, the PRO claims they also increasingly see the benefits of their products being reused, as it is an indication that the products are of high quality. The OEMs are also positive towards the positive social implications (e.g. job creation) associated with reuse. The PRO also envisions that they will offer more and more support for reusers that follow the guidelines, such as access to more information and repair manuals. Thus, the PRO encourages the emerging reuse business to become more professional.

In the future, the PRO sees potential further developments related to reuse and repairs, such as an increasing harvesting of spare parts for repairs from their EOL streams. They foresee that better use of data could provide ideas on the volumes of products and components on the market and when EOL products and/or reuseable spare parts may become accessible.

#### Norsirk Case

Norsirk, a PRO in Norway, works with an internal goal of 10% reuse. To achieve this, it works with dealers and municipal recycling stations and the social enterprise Sirkular, to increase reuse of collected white goods, in particular refrigerators, washing machines and stoves. The used products go through quality control and cleaning at Sirkular before they are placed on the market. Norsirk stated that public financial support for social enterprises was key to making the reuse business viable and for training the staff in Sirkular. The municipality operated the collection service, and this service was key to separating potentially reusable white goods. For this purpose, Norsirk developed a simple guide to aid municipalities. For example, appliances that are more likely to be reused are the ones who have newer or durable designs, larger front doors, intact wires, not obvious wear marks and a full array of removable parts (e.g. shelves). Products with older designs, small doors, cut wires, broken fronts, significant scratching or missing drawers/shelves are not separated for reuse.

Norsirk considers current and future EU regulations to be a main driver for the developments and also domestic policies related to the CE. They see an increasing interest from OEMs and their collective organisations, for reuse, and are working with them in further developing the practices of reuse. Interestingly, Norsirk views their reuse activities as a potential competitive advantage against other return schemes (PROs) in Norway. They think there will be increasing pressure on PROs to show that they work actively with reuse. Norsirk foresees some kind of certification scheme, with warranties and clear information on who to contact in case reused products are faulty, in the future, and are considering guidelines and other factors that must be in place. Norsirk also states that various public support—e.g. subsidised labour, or reduced rents for storage capacity—can be vital for supporting reuse practices.

## Discussion

The interview study largely confirms most of the barriers identified in literature. Even though preparation for reuse is technically in the PROs’ mission, they generally were perceived to lack incentives to engage in reuse of white goods. It was noted that the PROs found producers to be generally uninterested in engaging in reuse. There have been some suggestions in literature that OEMs in particular are against reuse and repair as it competes with sales of new products (‘cannibalisation’) [46], but this has been challenged in other studies [37] and in the case studies analysed in this contribution. This is a contradictory finding, as OEMs appear to be unwilling to invest in the repair or refurbishment of their own products, but they are concerned about the quality when third party actors are doing the repairs. One interviewee from a PRO suggested that OEMs selling high-quality brands are not too worried about cannibalisation, as it is mainly the cost-conscious consumers that buy reused products, so the competition is with low-cost brands.

Preparation for reuse is anticipated to become more prevalent within a gradual transition to a CE, and it is in OEMs interests to ensure profitability of these operations so that they maintain high quality (i.e. protect the OEM brand). One way to do this is by relying on highly qualified professional reuse businesses, but the question remains if these actors, once provided access to the waste stream, can achieve profitability in their operations.

The findings confirm that EPR schemes do focus more on recycling, while they do not find sufficient incentive to engage in reuse operations. This focus also means EOL products are treated as waste and are most often subject to rough transportation and exposure to weather elements, which deteriorates the physical properties of products (both aesthetically and functionally) and may reduce their reuse potential.

Key for PROs willingness to open up the waste stream is seemingly assurance of safety and high quality of the end result, so that no further liabilities are incurred in the OEMs—i.e. the members of the PROs. For this reason, PROs may only be willing to provide access to qualified professional reuse organisations. Limiting the access to the EPR waste stream only to these entities remains a barrier to non-professional reuse organisations, such as non-profit organisations, which do not have the same business models and profitability requirements as traditional reuse businesses. This may also hamper the potential of spare parts harvesting which is a secondary operation as it does not require the repair and refurbishment of entire appliances.

The case studies demonstrate that PROs in both Norway and Sweden are starting to work with reuse of white goods. There are several reasons behind this gradual shift but mostly due to circular economy trends both in policy and business and due to higher realisation that prospective product regulation might follow. Additionally, expanding reuse opportunities in EPR waste streams (such as in the case of EOL white goods) could be economically beneficial for the involved parties but also beneficial for the environment and wider society. This way, PROs not only enhance the environmental and social performance of their member OEMs but also could secure more funding opportunities for their operations in general, through business relations with reusers and potentially through public funding (subsidies), as most of the interviewees have suggested it to be a strong driver for increased reuse. In the cases examined, working with reuse was motivated by recognition that reuse would increasingly be a focus of the WEEE Directive in the future. However, reuse involves costs for separation, training, and preparation, skills that were not covered by the PRO funding. In the Norwegian case, these costs were addressed through partnering with social enterprises, which were subsidised by the state.

Even when costs are addressed, there still can be challenges with returning reused items to the market. In one case study discussed above, the reconditioning of the products was done in Scandinavia, but due to high costs of labour and high costs for retailing, the products were exported and sold in a Balkan country. This is an example of the problem with reuse of white goods: it is a low-margin sector, where sales mainly compete with sales of cheap new products. Thus, consumers do not pay a premium for reused products, but instead expect a significant reduction in price compared to new products, combined with a reasonable warranty. As OEMs typically expect higher profit margins, it is understandable that they are hesitant to enter this market.

Repair and refurbishment of EOL products are intrinsically related to high costs due to the number of labour hours required to perform repair activities which are essentially non-automatised and quite specific processes. Moreover, the transport of large volumes and weight of EOL white goods (fridges, kitchen ovens, etc.) and high warehousing requirements (spatially and temporally) raise the costs even more. Through the case examples, several strategies were identified to contain the costs related to reuse operations. While in the first PRO example, the export of reused products in lower cost countries contributed in keeping the overall costs low and the profit margin acceptable; in the second case of the Norwegian PRO, the solution was found through public-private partnerships and the social economy.

Table 3 provides an overview of the barriers and drivers identified.

**Table 3:** Criteria for using EOL products from EPR streams

Process phase	Barrier	Driver
Overall	Lack of reuse target	Policies and targets for reuse
	Lack of producer collaboration, due to their linear business models & concern about quality, brand value & liability	
		Mandate safety re-testing of used goods
	Unwillingness from PROs to pay, requiring operation to be profitable	Some actors can make the operation profitable
		Government subsidies
Collection, transportation and separation for preparation for reuse	Not all consumers take appliances to collection point	Deposit system paid to seller of new product and returned when taking appliance to waste collection point
	Collection actors lack of incentives to check reusability & handle with care	Legislate sorting and careful handling
		Create separate channel for reusable WEEE
		Allow access to third parties with incentives to check functionality and handle with care
	Access restricted by PRO	
	Collection actors and recyclers lack skilled staff for preparation for separation	
	High operational cost (incl. labour) & issues of profitability	High volumes & collaborations
		Combine reuse with other operations and job training
	Administrative burden (reporting)	
Repair & Refurbishment in Preparation for Reuse	High diversity of WEEE	Standardisation of tools, components and design
	Product not designed for reparability	More modular, repairable design
	Lack of access to necessities & cost -cost competitive access	Mandate OEMs to provide access
		Improve harvesting of spares
	High labour cost and lack of skilled labour	
	PRO/producer concerns about brand risk	Restrict preparation for reuse to professional 3rd parties
	Legal barriers, e.g. IP laws	
Reuse	Limited consumer demand	Information on ecological impact
	Consumers have low willingness to pay & lack of trust (brand & age)	Quality assurance, such as warranty
	Cannibalisation of sales of new	
	Movement of reused good	

There is a tension between increasing access for reusers to EPR streams and ensuring quality of reuse process: poor repair/refurbishment practices would result in a risk for brand reputation. While the solution to restrict access to professionals helps, it also hinders non-profit organisations from accessing the waste. Further, PROs and OEMs want reusers to provide good warranties and to follow certain standards. This of course increases the price for reused products but makes sense as it is important both to ensure a certain quality so that consumers have a good perception of reused products and in order to reduce the risks for brand reputation for OEMs. Further, reuse of ICT is a much larger market, with much better profit margins, and ICT remanufacturers often offer a 1-year warranty and sometimes offer the customer to purchase a 3-year warranty.^1^ It seems reasonable that reusers of white goods should strive towards similar practices.

Concluding the discussion, and taking into account the interview findings and the case studies, Table 4 presents a set of proposed interventions for upscaling the preparation for reuse of WEEE (white goods). The interventions are grouped into process- and product-related operations, as well as top-down policy interventions. Both approaches are required to address the multiple barriers of reuse and to incentivise PROs and other reuse actors to engage further in increasing the repair and reuse of EOL products. It should be noted that the suggestion to ‘Ban the throwaway of functioning products into EOL streams’ cannot be implemented without a proper investigation of the feasibility and appropriateness of such measures.

**Table 4:** Proposed interventions to support the upscaling of preparation for reuse

Product- and process-level (bottom-up approach)	Policy-level (top-down approach)
-Clear guidelines for reusers on conditions for reuse -Reusers should re-label and offer a warranty for the reuse products, as this would make OEMs less concerned about their responsibilities -Standardise and ‘modularise’ components and product design	-A mandatory reuse target in the WEEE Directive -Financial support from the government to the reuse sector -Ban the throwaway of functioning products into EOL streams

## Conclusion

This study has assessed the main barriers and drivers for reuse of white goods from EPR schemes. The main findings related to drivers and barriers are in line with the previous research. The main barriers include the limited economic viability of the reuse sector and the need for large volumes of EOL products in order to make a profit, the low cost of new products and the high cost of labour. The main drivers appear to be the current and future CE policies, whereas consumer demand is quite limited. The case studies indicate that PROs want to engage with reuse and even see reuse activities as a potential source of competitive advantage in the future. They are currently drafting criteria that reusers must follow in order to get access to EPR streams and for safeguarding the interest of OEMs in the process.

Some previous studies have indicated that OEMs are resistant to reuse activities as they may cannibalise on sales of new products, but generally, it appears that OEMs are starting to become more accepting of reuse of their products, provided certain conditions apply. The main reason for this is probably that reuse does not compete much with sales of premium products, but primarily competes with sales of low cost products. The fact that one Scandinavian reuser sells the reused products in a third country is also an indication that there is little direct competition with sales of new products in the domestic market.

The two case studies indicate that PROs are getting more engaged in reuse activities, and the draft guidelines assessed provide some ideas on what criteria are crucial for the PROs and its OEM member companies if they are to accept and encourage reuse of their products. The reuse should be done on a commercial basis, and reusers should bear the main responsibility for the reused products. OEMs will have the right to recall certain products, and precautionary measures must be taken so the reuse activities cannot lead to illegal waste export of WEEE.

The main contribution of this research has been the possibility to further explore various themes in the literature through an interview study. The research has confirmed some previous findings but also contributed to new knowledge. For instance, it is clear that PROs experience drivers to support reuse even in the absence of legal drivers, and we have a better picture of the criteria they will use to screen reusers. We also notice that OEMs are not very concerned about ‘cannibalisation’, at least not in a Scandinavian context.

Further research could develop in several directions. There is a need for more studies related to the reuse industry per se. Further, since private consumers do not drive the markets for used white goods and do not pay a premium for reused goods, there is potential for the public sector and large buyers (e.g. housing associations) to support reused products through their procurement. The public sector is increasingly buying reused computers and furniture [3, 55], and there could be potential in additional product categories.

## Data Availability

N/A

## Footnotes

1 One example is Inrego, the largest ICT remanufacturer in Scandinavia: inrego.se.

## Code Availability

N/A
